# Single Nucleotide Polymorphisms Associated with AA-Amyloidosis in Siamese and Oriental Shorthair Cats

**DOI:** 10.3390/genes14122126

**Published:** 2023-11-25

**Authors:** Stella L. Esders, Kirsten Hülskötter, Tom Schreiner, Peter Wohlsein, Jessica Schmitz, Jan H. Bräsen, Ottmar Distl

**Affiliations:** 1 Institute for Animal Breeding and Genetics, University of Veterinary Medicine Hannover (Foundation), 30559 Hannover, Germany; stella.esders@tiho-hannover.de; 2 Department of Pathology, University of Veterinary Medicine Hannover (Foundation), 30559 Hannover, Germany; kirsten.huelskoetter@tiho-hannover.de (K.H.); tom.schreiner@tiho-hannover.de (T.S.); peter.wohlsein@tiho-hannover.de (P.W.); 3 Nephropathology Unit, Institute of Pathology, Hannover Medical School, 30625 Hannover, Germany; schmitz.jessica@mh-hannover.de (J.S.); braesen.jan@mh-hannover.de (J.H.B.)

**Keywords:** AA-amyloidosis, Siamese cat, Oriental shorthair cat, genome-wide association study

## Abstract

AA-amyloidosis in Siamese and Oriental shorthair cats is a lethal condition in which amyloid deposits accumulate systemically, especially in the liver and the thyroid gland. The age at death of affected cats varies between one and seven years. A previous study indicated a complex mode of inheritance involving a major locus. In the present study, we performed a multi-locus genome-wide association study (GWAS) using five methods (mrMLM, FASTmrMLM, FASTmrEMMA, pLARmEB and ISIS EM-BLASSO) to identify variants associated with AA-amyloidosis in Siamese/Oriental cats. We genotyped 20 affected mixed Siamese/Oriental cats from a cattery and 48 healthy controls from the same breeds using the Illumina Infinium Feline 63 K iSelect DNA array. The multi-locus GWAS revealed eight significantly associated single nucleotide polymorphisms (SNPs) on FCA A1, D1, D2 and D3. The genomic regions harboring these SNPs contain 55 genes, of which 3 are associated with amyloidosis in humans or mice. One of these genes is *SAA1*, which encodes for a member of the Serum Amyloid A family, the precursor protein of Amyloid A, and a mutation in the promotor of this gene causes hereditary AA-amyloidosis in humans. These results provide novel knowledge regarding the complex genetic background of hereditary AA-amyloidosis in Siamese/Oriental cats and, therefore, contribute to future genomic studies of this disease in cats.

## 1. Introduction

Amyloidosis is a condition characterized by the deposition of amyloid, an insoluble, proteinaceous substance, that accumulates in various organs and tissues [1]. These deposits cause an impairment of organ architecture via pressure atrophy of adjacent cells, thus potentially causing loss of function of affected organs [1]. Amyloid possesses a fibrillary architecture and a ß-pleated sheet conformation, which is responsible for the affinity for Congo red stain and green birefringence in polarization microscopy [2]. In cats, a systemic form of amyloidosis is Amyloid A amyloidosis (AA-amyloidosis), with acute phase reactant Serum Amyloid A (SAA) as its precursor protein [3,4]. The predisposed breeds are Abyssinian and Somali along with Siamese and Oriental, in which hereditary forms of AA-amyloidosis are recognized [5,6,7,8]. In Abyssinian/Somali cats, the continuous failure of the kidneys until the age of five years due to a high degree of medullar and glomerular amyloid deposits is the main cause of death of affected cats [5,9,10]. In Siamese/Oriental cats, the organs mostly affected by the deposition of amyloid are the liver and the thyroid gland, but variable other organs and tissues can be affected as well [11]. The main cause of death of affected cats is rupture of the liver with subsequent blood loss in the abdominal cavity and hypovolemic shock [6,7,8,12]. The disease is lethal with a variable age at death between one and seven years [6]. Hereditary AA-amyloidosis in Oriental cats is autosomal, and neither the onset nor progression of the disease is influenced by the sex [11]. A segregation analysis of 17 clinically affected cats revealed a complex mode of inheritance, composed of a polygenic component with a major locus with Mendelian inheritance and heritability estimates based on the pedigree data of 0.56 ± 0.09 [11].

A study comparing Amyloid A protein sequences of one affected Abyssinian cat and two affected Siamese cats concluded that sequence differences may contribute to the different tissue affinities in these breeds, but this conclusion was not confirmed in past studies [13]. A recent study of two affected Abyssinian cats and 195 controls from different breeds concluded a polygenic background of AA-amyloidosis in this breed and found variants in *Amyloid Beta Precursor Protein gene (APP)* and *Transthyretin (TTR)* in affected cats [14]. Mutations in *APP* and *TTR* are associated with amyloidosis in humans [15,16,17,18].

The objectives of the present study were to identify variants associated with hereditary AA-amyloidosis in mixed Siamese/Oriental cats based on Illumina Infinium Feline 63 K iSelect DNA array data of cases and controls with multi-locus GWAS models. Within the genomic regions of these variants, we searched for putative functional candidate genes that have previously been reported to be associated with hereditary forms of amyloidosis in humans and mice [15,18,19,20].

## 2. Materials and Methods

### 2.1. Ethical Statement

All animal work was conducted following the national and international guidelines for animal welfare following the guidelines of the Declaration of Helsinki and was approved by the Institutional Review Board of the University of Veterinary Medicine Hannover (Foundation) and the responsible state veterinary office of Lower Saxony (registration number 33.9-42502-05-18A284). The sampling and handling of the animals followed European Union guidelines for animal care and handling and the Guidelines of Good Veterinary Practices. Cats were euthanized based on a thorough veterinary diagnosis and then submitted to the Department of Pathology, University of Veterinary Medicine Hannover (Foundation), by cat owners. EDTA-blood, hair root and buccal swab samples of controls were obtained from the biobank of the Institute for Animal Breeding and Genetics, University of Veterinary Medicine Hannover, and were taken for diagnostic purposes by accredited veterinarians.

### 2.2. Animals

In the present study, we included 68 cats mainly collected from a German cattery consisting of mixes between Siamese and Oriental shorthair cats previously analyzed in the complex segregation analysis [11] (Supplementary Figure S1 and Table S1).

Cases include cats that were affected by clinical signs consistent with systemic AA-amyloidosis as rupture of the liver and died between the ages of one and seven years (*n* = 20). All cases belong to the aforementioned cattery. Histopathological confirmation of the diagnosis was available for 16/20 cats. Cats from the same breeds that never showed clinical signs consistent with systemic AA-amyloidosis and were at least ten years old at the time of the analyses were used as controls (*n* = 48). Relationships of the cats are shown in Supplementary Figure S1. Entry numbers 67 and 68 are not shown in Supplementary Figure S1 as they are not closely related to the other cats (1–66) in this study. 

### 2.3. Histological and Immunohistochemical Examination

Histological and immunohistochemical examinations were performed as previously described [11]. Briefly, the tissue of the 16 autopsied cats was fixed, stained with Congo red and spectated via polarization light microscopy to confirm amyloid deposits. The localization of amyloid deposits in different organs and tissues was evaluated (Supplementary Table S2).

Additionally, in 9/16 cases, distributed across the different generations with affected individuals, amyloid classification was performed. In these nine animals, Amyloid A was classified with immunostaining, which was conducted with an automated platform (Ventana ULTRA; Ventana Medical Systems) with mouse monoclonal antibodies [11].

### 2.4. Genotyping on the Illumina Infinium Feline 63 K iSelect DNA Array

Genomic DNA was isolated from EDTA-blood samples, buccal swabs and hair roots of the 48 controls, and tissue samples of the 20 cases using phenol/chloroform extraction. DNA concentration per sample was adjusted to 50 ng/µL with Nanodrop ND-1000 (Peqlab Biotechnology, Erlangen, Germany). Genotyping for the 68 cats was performed using the Illumina Infinium Feline 63 K iSelect DNA array with 62,897 SNPs (Neogen, Lincoln, NE, USA). Chromosomal positions of the SNPs were identified using the current reference genome from the National Center for Biotechnology Information (NCBI) F. catus_Fca126_mat1.0 (https://www.ncbi.nlm.nih.gov/datasets/genome/GCF_018350175.1/, accessed on 4 September 2023) [21] and BLAST [22].

### 2.5. Genome-Wide Association Studies

The genome-wide association analyses were performed using the R package mrMLM v4.0.2 [23], which implements five methods: the multi-locus random-SNP-effect mixed linear model (mrMLM) [24], the fast multi-locus random-SNP-effect mixed linear model (FASTmrMLM) [25], the fast multi-locus random-SNP-effect efficient mixed model association (FASTmrEMMA) [26], the polygenic-background-control-based least angle regression plus empirical Bayes (pLARmEB) [27] and the iterative sure independence screening expectation-maximization Bayesian least absolute shrinkage and selection operator (ISIS EM-BLASSO) [28].

In single-locus GWAS, usually the Bonferroni correction is applied. The Bonferroni correction incorporates the number of tests by dividing the significance threshold by the number of tests and is used in multiple testing scenarios to reduce the type I error since the likelihood of type I errors increases as the number of tests increases [29]. In complex traits, using single-locus methods bears the risk of missing associated SNPs since they do not surpass the significance threshold [30]. The implementation of multi-locus models in GWAS can help overcome this risk since there is no need for the often excessively conservative Bonferroni correction because of the multi-locus nature of these methods considering every SNP simultaneously [24]. In multi-locus GWAS, the analyses are conducted in two stages. In the first stage, an initial genome-wide single-locus screening selects all SNPs that surpass a moderate significance threshold of 0.01 or 0.005, depending on the multi-locus method. In the second stage, multi-locus models are implemented only for the subset of SNPs that passed the initial screening stage, and SNP effects are estimated simultaneously [26,28]. For the second stage, a significance threshold for the logarithm of the odds (LOD) score of 3 was applied, which is recommended based on the current literature [28]. The study design reached enough power for the detection of significant loci when loci are in high linkage disequilibrium with the potential causative mutation. The perfect association enables reaching *p*-values of 1.714 × 10^−15^, even when 4, 6 and 8 cases are misclassified by the markers *p*-values of 1.247 × 10^−11^, 6.49 × 10^−10^ and 2.50 × 10^−8^ are obtained, respectively (Supplementary Table S3).

Data quality control was performed using PLINK v.1.9 (www.cog-genomics.org/plink/1.9/, accessed on 1 August 2023) [31]. Applied quality criteria included minor allele frequency (MAF) of >0.05 and genotyping rate per SNP and animal of >0.9. Of the 39,246 SNPs that met these quality criteria, only SNPs identified by at least two of the five applied multi-locus methods were included in the putative candidate gene search. Manhattan and Q-Q plots were generated using the calculated −log_10_ *p*-values from the multi-locus GWAS using the mrMLM v4.0.2 package in R software, version 4.1.1.

### 2.6. Putative Candidate Gene Identification

Linkage Disequilibrium (LD) for significantly associated SNPs was calculated using the −r2 command from PLINK 1.90 (www.cog-genomics.org/plink/1.9/, accessed on 1 August 2023) [31], and a threshold of >0.7 for squared correlation between a SNP and another SNP within the 1 Mb region was applied.

Genes within these genomic regions were screened using the current reference genome from NCBI F. catus_Fca126_mat1.0. (https://www.ncbi.nlm.nih.gov/datasets/genome/GCF_018350175.1/, accessed on 4 September 2023) [21]. Putative candidate genes were functionally annotated according to the GeneCards database [32] and literature. The determined genes were further compared to a list of genes associated with AA-amyloidosis from NCBI (https://www.ncbi.nlm.nih.gov/gene/?term=AA-amyloidosis, accessed on 4 September 2023). Furthermore, we compared the results of the present study to the results of the multi-omic study in Abyssinian cats [14]. Potential networks between putative candidate genes and all genes in the region with LD to the associated SNP were analyzed using GeneMANIA [33].

## 3. Results

### 3.1. Multi-Locus GWAS

**Figure 1:**
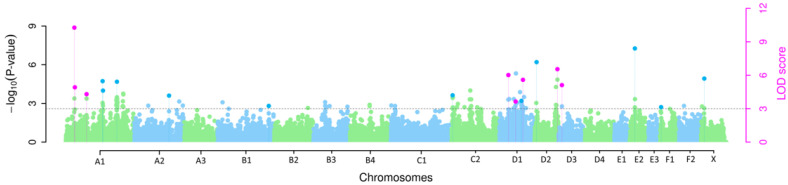
Manhattan plot of *p*-values and LOD-scores for AA-amyloidosis in mixed Siamese/Oriental cats. Each dot represents one SNP of the multi-locus GWAS. Blue and green dots represent −log_10_ *p*-values of SNPs identified by one multi-locus method. The genome-wide −log_10_ *p*-values and LOD scores for each SNP effect are plotted against their chromosomal position. Pink dots represent SNPs identified by at least two of the applied methods with LOD scores > 3.

The multi-locus GWAS revealed eight SNPs with a significant association with hereditary AA-amyloidosis in mixed Siamese/Oriental cats with LOD scores > 3 that were detected using at least two multi-locus methods simultaneously (Figure 1 and Table 1). All SNPs that exceeded the LOD scores > 3 were only detected using one multi-locus method, which are provided in Supplementary Table S4.

The method FASTmrMLM detected five of the eight significant SNPs, whereas mrMLM, FASTmrEMMA, ISIS EM-BLASSO and pLARmEB detected four of the eight significant SNPs each (Supplementary Table S5). Of the eight identified SNPs, three are located on *Felis catus* chromosome (FCA) A1, three on FCA D1 and one each on FCA D2 and FCA D3. Two of the significantly associated SNPs on FCA A1 are located within an interval of 1.5 Mb. The most significantly associated SNP is CHRA1.33307498 on FCA A1 (LOD score 5.40–12.65), which also explains the highest proportion of the phenotypic variance in the mrMLM (26.61%), FASTmrMLM (28.50%) and FASTmrEMMA (16.75%) methods. For pLARmEB, the SNP CHRA1.35096216 explains the highest proportion of the phenotypic variance at 17.36% and the ISIS EM-BLASSO CHRD2.113529492 at 27.57%. The SNP identified by most multi-locus methods (*n* = 4; FASTmrMLM, FASTmrEMMA, pLARmEB and ISIS EM-BLASSO) is CHRD2.113529492 on FCA D2.

The phenotypic variance estimated for the trait is 0.2067 (Supplementary Table S5). The five multi-locus methods, mrMLM, FASTmrMLM, FASTmrEMMA, pLARmEB and ISIS EM-BLASSO explained 93.55%, 65.05%, 40.58%, 87.74% and 83.91% of the phenotypic variance, respectively, when considering every identified SNP (Supplementary Table S6).

A quantile–quantile plot (Q-Q-plot) shows the observed *p*-values plotted against the expected *p*-values under the null hypothesis (Supplementary Figure S2). The distribution of AA-amyloidosis-affected Siamese/Oriental cats by their genotypes of the significantly associated SNPs is shown in Supplementary Table S7. The susceptible genotype of four SNPs explained more than 60% of the cases when at least ten cases were present for the most associated genotype. By combining the four most strongly associated genotypes into a concatenated genotype, the phenotypes of all but three animals were correctly assigned.

### 3.2. Candidate Gene Search

The 1 Mb upstream and downstream of the identified genomic regions significantly associated with SNPs were screened and 55 genes in these regions were functionally annotated (Supplementary Table S8). Two of the significantly associated SNPs (CHRUN5.1424878 and CHRD1.112359805) are located within introns of genes (*IGSSF9B* and *NAV2*), whereas the other SNPs are located in intergenic regions. Functional annotation of these genes provided eight putative candidate genes in close vicinity to three of the significantly associated SNPs (Table 2). 

Among the genes retrieved in LD with associated SNPs, *SAA1* was identified, which encodes for the precursor of Amyloid A and thus may be a putative candidate gene. Two other putative candidate genes, namely *SLC10A2* and *ZDHHC13*, have been reported to be associated with amyloidosis in humans and/or mice in previous studies (Table 2). *ZDHHC13* may be a stronger putative candidate as it plays a role in thyroid gland disease in mice and the thyroid gland is one of the main target organs of AA-amyloidosis in Siamese and Oriental shorthair cats. Furthermore, a genomic region on FCA D1 harboring four genes (*HPS5*, *GTF2H1*, *LDHA* and *LDHC*) may be considered relevant as this region has a major impact on the precursor of Amyloid A, SAA, in humans (Table 2). The analysis of the genetic interactions of the genes within the 1 Mb genomic regions surrounding the significantly associated SNPs and the eight putative candidate genes revealed shared protein domains, illustrated with green lines, as well as physical interactions, illustrated with red lines, and co-expressions, illustrated with purple lines (Supplementary Figures S3 and S4). 

## 4. Discussion

We identified eight significantly associated SNPs on four cat chromosomes (FCA A1, D1, D2, and D3), confirmed by at least two approaches with mrMLM. The most consistent genomic region among the mrMLM methods employed was identified on FCA A1. Within this 1.5 Mb region, each of the five multi-locus methods identified one significantly AA-amyloidosis-associated SNP (CHRA1.33307498 or CHRA1.35096216). These two SNPs on FCA A1 explained 16.75–28.50% of the phenotypic variance, depending on the multi-locus method applied. Each multi-locus method also identified at least one of the three significantly associated SNPs on FCA D1 (CHRUN5.1424878, CHRD1.83154354 and CHRD1.112359805). The simultaneous detection of the same SNP which was found to be significantly associated with AA-amyloidosis by at least two multi-locus methods corroborates the likelihood of the association of the SNP with the trait. The results of the present study propose the involvement of several loci in the development of AA-amyloidosis in the investigated breeding population of mixed Siamese/Oriental cats. These findings are consistent with the results of the complex segregation analysis, according to which the inheritance of systemic AA amyloidosis is most likely not monogenic but genetically complex [11].

A recent report based on whole genome sequencing (WGS) data of cats affected by AA-amyloidosis concludes that AA-amyloidosis in Abyssinian cats is also not a monogenic trait [14]. Genome variant detection revealed 101 missense variants and one frameshift deletion as disease-associated when only considering the coding gene variants in this WGS study [14]. Following candidate gene search within these variants revealed *Amyloid Beta Precursor Protein (APP)* and *Transthyretin (TTR)* genes that have previously been reported to be associated with the deposition of amyloid in humans [14,15,17]. *APP* and *TTR* are not located within the genomic regions surrounding the significantly AA-amyloidosis-associated SNPs in the present study. Nevertheless, the putative candidate gene *PTPRE* is located within the genomic region of CHRD2.113529492 on FCA D2, and a single nucleotide variant in this gene was solely identified in affected Abyssinian cats [14]. *Protein Tyrosine Phosphatase Receptor Type E gene* (*PTPRE)* encodes for a member of the protein tyrosine phosphatase family, which regulates various different cellular processes such as cell differentiation and oncogenic transformation [37]. This gene has further been reported to play a role in different forms of cancer including thyroid carcinoma [36]. The thyroid gland was affected by amyloid deposition in 100% of the cases in the present study and in one Abyssinian cat in the multi-omic study, thus a somehow compromised background of the thyroid gland might play a role in the affectedness of this organ. Nevertheless, *PTPRE* has not been reported in the context of amyloidosis and thus this assumption is speculative. Future analyses of the WGS data of identified genomic regions should nonetheless include this gene to explore a possible link between *PTPRE* and systemic AA-amyloidosis. Concordant with the multi-omic analyses performed in Abyssinian cats, we consider that a compromised organic background of the main target organ could facilitate the deposition of amyloid or might play a role in the differences in the mainly affected organs in the different cat breeds [14]. Even though there is a possibility of a common genetic background of AA-amyloidosis in Siamese/Oriental cats, Abyssinian cats and other feline species, it is also important to keep in mind that the genetic background could differ between these breeds and species.

It has previously been shown that human chromosome 11 and FCA D1 are syntenic for large segments [38]. A region within 1 Mb one of the significantly associated SNPs (CHRD1.112359805) on FCA D1 harbors the same genes, including *HPS5*, *GTF2H1*, *LDHA* and *LDHC*, as the region 11p15.5-p13 in humans. The region 11p15.5-p13 has a major impact on SAA, the precursor protein of Amyloid A, and thus represents a highly relevant genomic region for hereditary AA-amyloidosis in cats [35]. This region also harbors *Serum Amyloid A1 (SAA1)*, an important putative candidate gene for AA-amyloidosis. A mutation in the *SAA1* promoter causes a hereditary form of AA-amyloidosis in humans; thus, *SAA1* could be of high importance in AA-amyloidosis in cats [19]. Nevertheless, a potential involvement of *SAA1* in hereditary AA-amyloidosis in cats has not been reported yet. The WGS data of *SAA1* need to be the focus of future investigations to review if *SAA1* is directly involved in hereditary AA-amyloidosis in cats.

Approximately 340 kb downstream of CHRD1.112359805, the gene *Zinc Finger DHHC-Type Palmitoyltransferase 13* (*ZDHHC13)* is located. The encoded enzyme modifies proteins via palmitoylation, which has been shown to be an important regulator of protein trafficking and protein stability [39]. Interestingly, a mutation in *ZDHHC13* caused a severe phenotype in mice including systemic AA- and AL-amyloidosis. Therefore, a variant in *ZDHHC13* might also play a role in systemic AA-amyloidosis in cats [20].

In other forms of amyloidosis, such as Aβ–amyloidosis in Alzheimer’s disease (AD), instead of a single gene, multiple genes mostly influence the pathogenesis.

Around 510 kb downstream of CHRA1.87876182 *Solute Carrier Family 10 Member 2 (SLC10A2)* is located. *SLC10A2* encodes a sodium/bile acid cotransporter and is a crucial factor for cholesterol homeostasis [40]. A SNP in close vicinity to *SLC10A2* is associated with late-onset Alzheimer’s disease (LOAD) in humans [34]. The potential role in the pathogenesis of LOAD has not been verified yet. Since this gene could be involved in the Aβ-amyloid accumulation in AD patients and not in AA-amyloidosis, drawing comparisons between these conditions is difficult. Nevertheless, the common occurrence of deposits of misfolded, insoluble proteins may be the result of some common features in the development of these conditions and thus justifies a closer look into genes possibly involved in Aβ-amyloid accumulation. 

Histopathological confirmation of the clinical diagnosis of accredited veterinarians was available in 16/20 cases. The remaining four cases were closely related to the cases with histopathological confirmation and the similarities in age at death due to clinical signs of amyloidosis corroborate the assumption of the presence of hereditary AA-amyloidosis in these cats.

The results of the present study need to be assessed in a larger cohort of other Siamese/Oriental cats from different populations and cats from other breeds to evaluate if the results may be valid in other Siamese/Oriental breeding populations, or if the findings can solely be confirmed in individuals belonging to the investigated cattery. Inbreeding coefficients were tested for association with the amyloidosis status in the same population. However, we came to the conclusion that inbreeding effects were not significant [11]. In the present study, we calculated genomic inbreeding based on Runs of Homozygosity, but we were not able to show significant effects. Variants that explained a large amount of phenotypic variance should be validated to see if the genomic regions harboring these variants show a significant risk of developing AA-amyloidosis in other Siamese/Oriental cats. If this is the case, searching for further variants in these genomic regions seems advisable.

Depending on the multi-locus method, GWAS results from the present study were able to explain a large amount of the phenotypic variance, but not all, which might have occurred since we excluded SNPs from putative candidate gene searches that did not reach the significance threshold. A limitation of GWAS in complex polygenic traits is that SNPs that do not surpass the significance threshold could nevertheless be associated with the investigated trait if their effect is small. Such variants could play a role in deciphering the genetic background of the trait. We aimed at reducing this risk by choosing different multi-locus methods, which have previously been proven more suitable for polygenic traits [30,41].

Considering this, further work should explore genomic regions that were identified in the present study with WGS data and screen for variants involved in AA-amyloidosis in Siamese/Oriental cats. The regions explaining the highest amounts of phenotypic variance on FCA A1, D1 and D2 and the gene *SAA1* should be the focus of these investigations. Increased knowledge of the underlying genetic background in Siamese/Oriental cats will help decipher the pathogenesis of the condition and enable genomic selection to reduce the prevalence of the lethal condition and further aid in identifying similarities and differences with other feline species. The limitations of this study are the low number of cases and controls as well as the origin of cases and controls from a smaller subpopulation of Siamese/Oriental cats. A sampling of cases and controls is difficult because cat breeders are not willing to provide euthanized animals for necropsy and donate samples from older and healthy cats. Due to these sampling limitations, the power to identify associated variants is reduced. Therefore, additional genetic and structural variants not detectable in these data may be relevant to AA amyloidosis. A further study with independent and more data is warranted to confirm these findings and to show whether a smaller number of genetic variants can capture more than 90% of the phenotypic trait variance. A similar approach to a previous report on Abyssinian cats [14], using cats of the genetically predisposed breed affected with AA amyloidosis and controls from a large sample of very different cat breeds not genetically disposed to AA amyloidosis, may also help to corroborate the present findings. The variants and genomic regions identified in this study may, therefore, be useful for further population studies to confirm possible associations. In addition, the identified putative candidate genes and variants may also be a source for screening whole-genome sequencing data and meta-analyses.

## 5. Conclusions

Hereditary AA-amyloidosis in mixed Siamese/Oriental cats is not a monogenic trait but a complex condition. The region harboring *SAA1*, *HPS5*, *GTF2H1*, *LDHA* and *LDHC*, which has a major impact on SAA in humans and the genomic regions that explain a large amount of the phenotypic variance, should be investigated in future studies based on WGS data of cases and controls to identify putatively causal variants for systemic AA-amyloidosis in Siamese/Oriental cats. Further studies with larger datasets are needed to elucidate the complexity of genetic and structural variants involved in AA-amyloidosis in Siamese/Oriental cats. 

## Figures and Tables

**Table 1:** SNPs significantly associated with hereditary AA-amyloidosis in mixed Siamese/Oriental cats and detected by at least two methods simultaneously in the multi-locus GWAS.

SNP-ID	FCA	Position	MAF	LOD Score	PVE (%)	Method
CHRA1.33307498	A1	29,635,172	0.36	12.65	26.61	mrMLM
			0.35	5.40	28.50	FASTmrMLM
			0.35	10.27	16.75	FASTmrEMMA
CHRA1.35096216	A1	31,080,887	0.29	3.78	17.36	pLARmEB
			0.29	6.05	18.54	ISIS EM-BLASSO
CHRA1.87876182	A1	74,262,275	0.39	7.21	12.22	mrMLM
			0.40	4.30	4.21	FASTmrMLM
			0.40	3.88	3.74	FASTmrEMMA
CHRUN5.1424878	D1	30,498,477	0.31	5.20	15.65	FASTmrMLM
			0.31	6.84	15.35	ISIS EM-BLASSO
CHRD1.83154354	D1	52,625,246	0.45	3.07	3.23	FASTmrEMMA
			0.45	4.20	8.47	pLARmEB
CHRD1.112359805	D1	75,099,182	0.23	6.34	11.86	mrMLM
			0.23	3.49	1.96	FASTmrMLM
			0.23	5.59	5.01	pLARmEB
CHRD2.113529492	D2	84,125,513	0.35	6.32	14.73	FASTmrMLM
			0.35	6.77	10.81	FASTmrEMMA
			0.35	6.29	16.56	pLARmEB
			0.35	13.35	27.57	ISIS EM-BLASSO
CHRA1.170535359	D3	12,578,042	0.44	3.32	5.39	mrMLM
			0.44	6.91	10.35	ISIS-EM-BLASSO

FCA: *Felis catus* chromosome according to F. catus_Fca126_mat1.0; Position: SNP position in base pairs according to F. catus_Fca126_mat1.0; MAF: minor allele frequency; LOD score: logarithm of the odds scores; PVE: percentage of phenotypic variance explained by multi-locus methods.

**Table 2:** Putative candidate genes for AA-amyloidosis in mixed Siamese/Oriental cats and their chromosomal position on *Felis catus* (FCA) chromosome according to F. catus_Fca126_mat1.0.

SNP-ID	FCA	Gene	Start	End	Related Functions
CHRA1.87876182	A1	*SLC10A2*	73,755,036	73,774,055	Late onset Alzheimer’s disease [34]
CHRD1.112359805	D1	*SAA1*	74,193,158	74,196,364	AA-amyloidosis [19]
		*HPS5* *GTF2H1* *LDHA* *LDHC*	74,220,513 74,280,919 74,338,285 74,353,740	74,280,965 74,318,855 74,349,667 74,397,837	Major impact on SAA [35]
		*ZDHHC13*	74,760,625	74,812,445	AA-/AL-amyloidosis [20]
CHRD2.113529492	D2	*PTPRE*	83,094,722	83,258,667	Thyroid carcinoma [36]

## Data Availability

Restrictions apply to the availability of these data. Data were obtained from cat owners and are available upon reasonable request from the authors with the permission of the cat owners.

## Supplementary Materials

The following supporting information can be downloaded at: https://www.mdpi.com/article/10.3390/genes14122126/s1, Figure S1. Pedigree of the mixed Siamese/Oriental cats. Figure S2. Quantile-quantile (Q-Q) plot of expected *p*-values versus observed *p*-values of the GWAS for hereditary AA-amyloidosis in Siamese/Oriental cats. Figure S3. GeneMANIA network of 55 genes located within 1 Mb upstream and downstream of significantly associated SNPs. Figure S4. GeneMANIA network of eight putative candidate genes. Table S1. Animals included in the multi-locus GWAS. Table S2. Localization of amyloid deposits. Table S3. Considerations for identifying significant SNPs in this study depending on the correlation coefficient (r) between a SNP locus that perfectly matches the disease-causing SNP locus and correlated SNP loci. Table S4. Significant SNPs of the multi-locus GWAS. Table S5. Significant SNPs detected by multi-locus methods, phenotypic variance and residual variance of the GWAS-SNPs. Table S6. Phenotypic variance explained by identified SNPs using multi-locus methods. Table S7. Distribution (%) of AA-amyloidosis-affected Siamese/Oriental cats by their genotypes of significantly associated SNPs. Table S8. Putative candidate genes located 1 Mb upstream and downstream of significant SNPs.
