# Supplementary material for: Single Nucleotide Polymorphisms Associated with AA-Amyloidosis in Siamese and Oriental Shorthair Cats
Source: Genes (Basel). 2023 Nov 25;14(12):2126. doi: 10.3390/genes14122126 (PMC10742459; doi:10.3390/genes14122126)
Supplement: Supplementary file 1 [file genes-14-02126-s001.zip › Figure_S2_QQPlot.pdf]

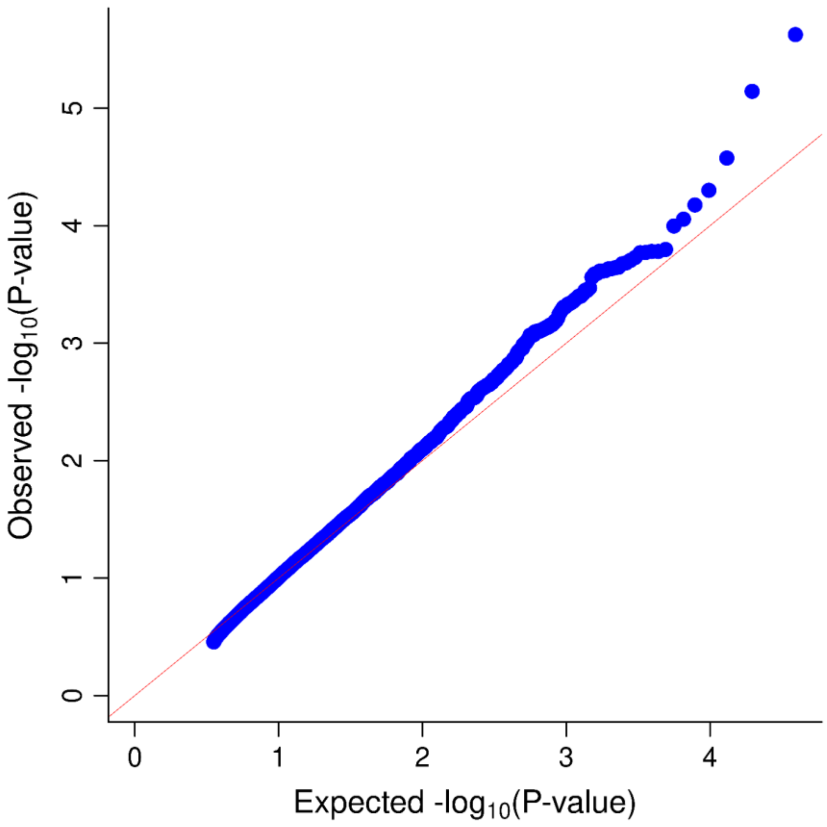

Figure S2. Quantile-quantile (Q-Q) plot of expected P-values versus observed P-values of the multi-locus GWAS for hereditary AA-amyloidosis in mixed Siamese/Oriental cats. The blue dots represent the included 39,246 SNPs and the red line indicates the distribution of the SNPs under the null hypothesis of no association.
