# Supplementary material for: Single Nucleotide Polymorphisms Associated with AA-Amyloidosis in Siamese and Oriental Shorthair Cats
Source: Genes (Basel). 2023 Nov 25;14(12):2126. doi: 10.3390/genes14122126 (PMC10742459; doi:10.3390/genes14122126)
Supplement: Supplementary file 1 [file genes-14-02126-s001.zip › Table_S3_Comparison of power to detect significant SNPs.pdf]

Table S3. Considerations for identifying significant SNPs in this study depending on the correlation coefficient (r) between a SNP locus that perfectly matches the disease causing SNP locus and correlated SNP loci.

Correlation coefficient	Genotype distribution						*p*-Value
	Genotypes of controls (n=48)			Genotypes of affected (n=20)			
	11	12	22	11	12	22	
1.0	0	24	24	20	0	0	1.714e-15
0.9566	0	24	24	18	1	1	1.747e-13
0.9146	0	24	24	16	2	2	1.247e-11
0.8740	0	24	24	14	3	3	6.490e-10
0.8345	0	24	24	12	4	4	2.500e-8
0.7960	0	24	24	10	5	5	7.760e-7
0.7583	0	24	24	8	6	6	1.883e-5
0.7212	0	24	24	6	7	7	3.719e-4
