# Supplementary material for: Single Nucleotide Polymorphisms Associated with AA-Amyloidosis in Siamese and Oriental Shorthair Cats
Source: Genes (Basel). 2023 Nov 25;14(12):2126. doi: 10.3390/genes14122126 (PMC10742459; doi:10.3390/genes14122126)
Supplement: Supplementary file 1 [file genes-14-02126-s001.zip › Table_S5_phenotypic_variance_residual_variance.pdf]

Table S5. Number of significant SNPs detected by multi-locus methods, estimates of total phenotypic variance and GWAS-SNP residual variance of hereditary AA-amyloidosis in mixed Siamese/Oriental cats.

Method	Number of SNPs	Phenotypic variance	GWAS-SNP-residual variance
mrMLM	4	0.2067	0.0183
FASTmrMLM	5	0.2067	0.0366
FASTmrEMMA	4	0.2067	0.0424
pLARmEB	4	0.2067	0.034
ISIS EM-BLASSO	4	0.2067	0.0294
