# Supplementary material for: Single Nucleotide Polymorphisms Associated with AA-Amyloidosis in Siamese and Oriental Shorthair Cats
Source: Genes (Basel). 2023 Nov 25;14(12):2126. doi: 10.3390/genes14122126 (PMC10742459; doi:10.3390/genes14122126)
Supplement: Supplementary file 1 [file genes-14-02126-s001.zip › Table_S6_PVE.pdf]

Table S6. Phenotypic variance explained (PVE) by identified SNPs by multi-locus methods associated with hereditary AA-amyloidosis in mixed Siamese/Oriental cats.

Method	SNP ID	PVE SNP	Total PVE of all detected SNPS
mrMLM	**CHRA1.33307498**	26.61	93.55
	**CHRA1.87876182**	12.22	
	CHRB1.204283203	7.55	
	**CHRD1.112359805**	11.86	
	CHRD2.3894331	11.55	
	**CHRA1.170535359**	5.39	
	CHRE2.26012148	10.75	
	CHRF1.2996541	7.62	
FASTmrMLM	**CHRA1.33307498**	28.50	65.05
	**CHRA1.87876182**	4.21	
	**CHRUN5.1424878**	15.65	
	**CHRD1.112359805**	1.96	
	**CHRD2.113529492**	14.73	
FASTmrEMMA	**CHRA1.33307498**	16.75	40.58
	**CHRA1.87876182**	3.74	
	CHRA1.169994536	6.05	
	**CHRD1.83154354**	3.23	
	**CHRD2.113529492**	10.81	
pLARmEB	**CHRA1.35096216**	17.36	87.74
	CHRA1.168026137	7.98	
	CHRA1.223820842	7.01	
	CHRA2.179873843	9.23	
	**CHRD1.83154354**	8.47	
	CHRD1.104974246	16.12	
	**CHRD1.112359805**	5.01	
	**CHRD2.113529492**	16.56	
ISIS EM-BLASSO	**CHRA1.35096216**	18.54	83.91
	CHRC2.4289851	5.71	
	**CHRUN5.1424878**	15.35	
	**CHRD2.113529492**	27.57	
	**CHRA1.170535359**	10.35	
	CHRX.13778263	6.39	

SNPs in bold have been detected by at least two methods simultaneously, PVE SNP: Percentage of phenotypic variance explained of each SNP identified by method, Total PVE: Percentage of phenotypic variance explained of all SNPs identified by method.
