# Supplementary figures and images for: Single Nucleotide Polymorphisms Associated with AA-Amyloidosis in Siamese and Oriental Shorthair Cats
Source: Genes (Basel). 2023 Nov 25;14(12):2126. doi: 10.3390/genes14122126 (PMC10742459; doi:10.3390/genes14122126)

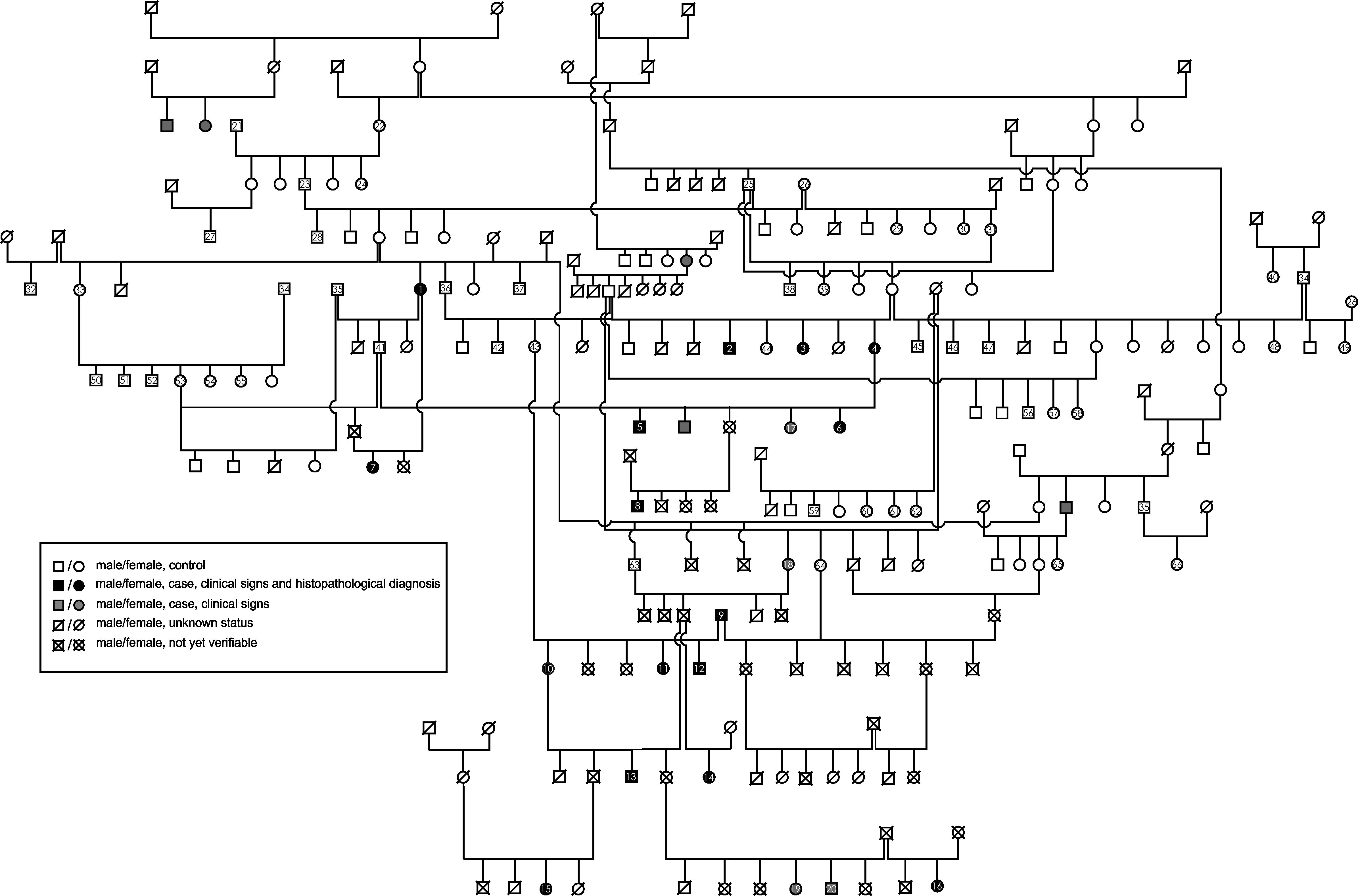

Supplement: Supplementary file 1 [file genes-14-02126-s001.zip › Figure_S1_Pedigree.jpg]

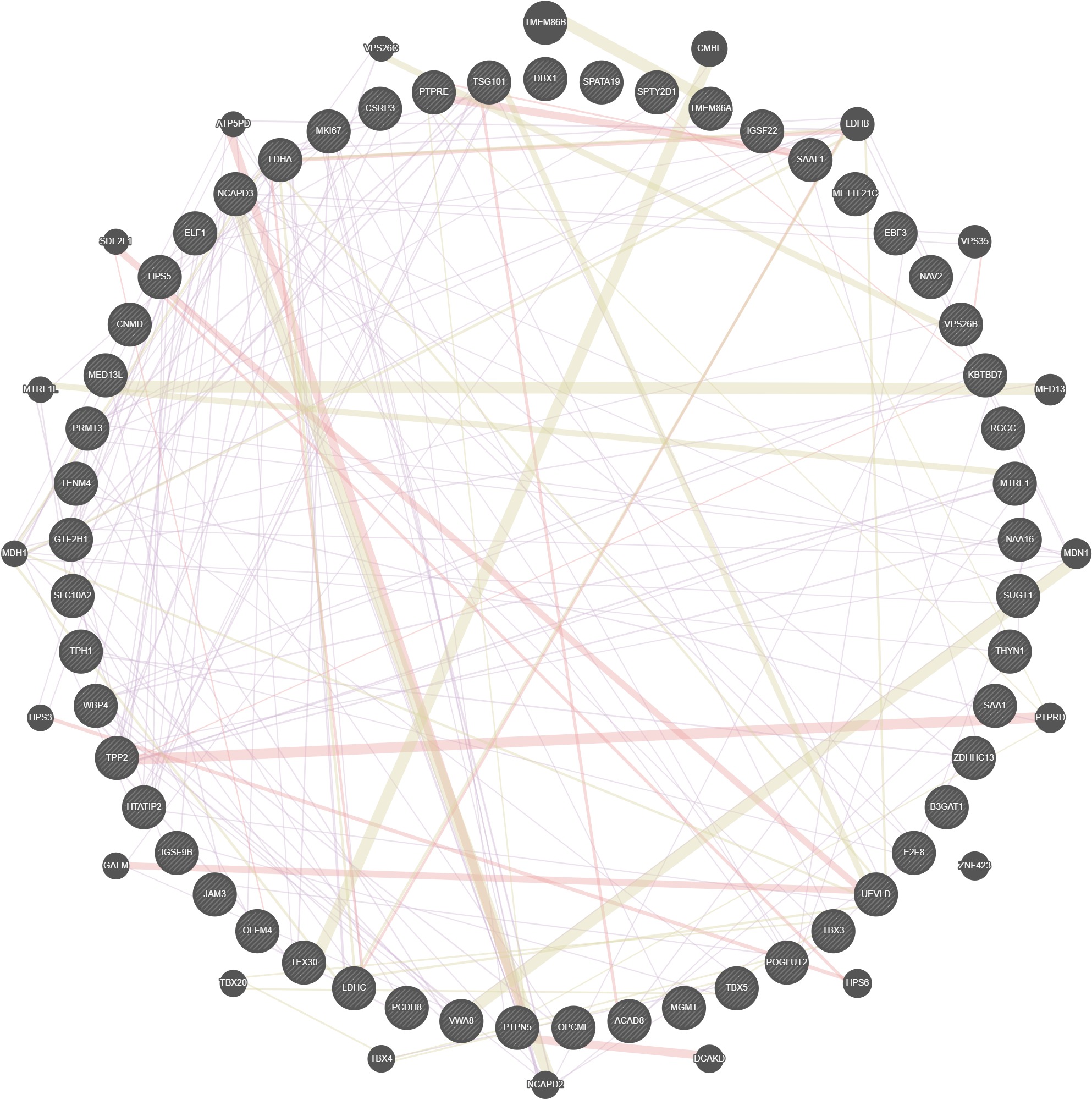

Supplement: Supplementary file 1 [file genes-14-02126-s001.zip › Figure_S3_genemania_network_55genes_in_genomic_regions.jpg]

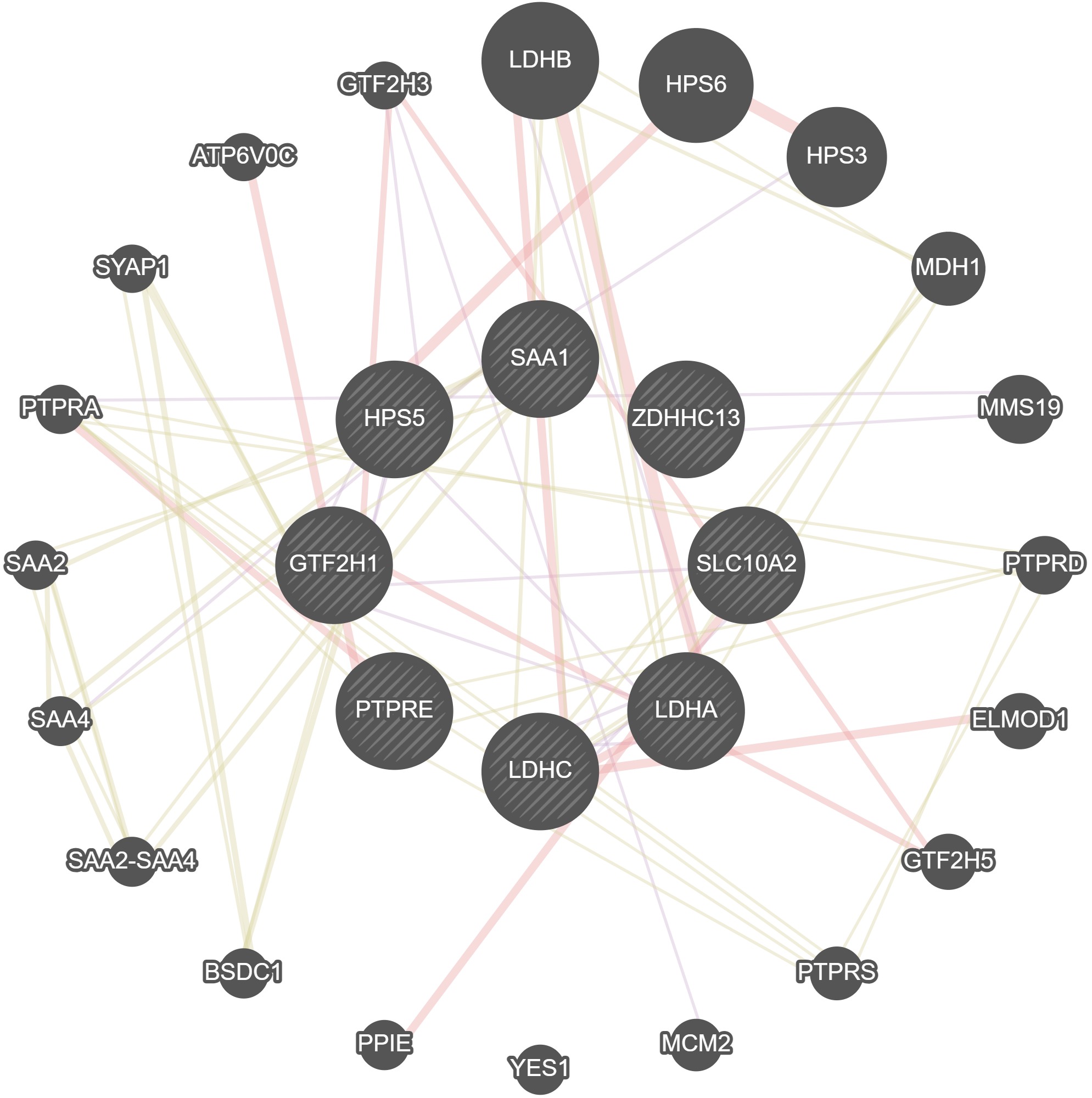

Supplement: Supplementary file 1 [file genes-14-02126-s001.zip › Figure_S4_genemania_network_8putative_candidate_genes.jpg]
